# Mitochondrial and plastid genome analysis of the marine red alga *Coeloseira compressa* (Champiaceae, Rhodophyta)

**DOI:** 10.1080/23802359.2016.1182452

**Published:** 2016-07-08

**Authors:** Zareen M. Kilpatrick, Jeffery R. Hughey

**Affiliations:** a Division of Science and Environmental Policy, California State University, Monterey Bay, Seaside, CA, USA;; b Division of Mathematics, Science, and Engineering, Hartnell College, Salinas, CA, USA

**Keywords:** Mitogenome, mtDNA, plastid genome, red algae, Rhodymeniales

## Abstract

The classification of species originally assigned to the marine red algal genus *Coeloseira* G.J. Hollenberg remains univestigated using molecular data. Paired-end 36 bp Illumina sequences (Illumina Inc, San Diego, CA) were generated from a specimen of *Coeloseira compressa* G. J. Hollenberg and used to assemble the partial mitochondrial and complete plastid genomes. The mitogenome is 25,391 bp in length and contains 46 genes, and the plastome is 176,291 bp with 233 genes. Both organellar genomes show high gene synteny with previously published Florideophyceae. Comparison of organellar and nuclear phylogenetic markers (*rbc*L, CO1, LSU) of *C. compressa* to other genera in the Champiaceae supports its removal from *Gastroclonium* Kützing and the reinstatement of the name *Coeloseira compressa*.

The red algal order Rhodymeniales is a marine taxon distributed worldwide that consists of six families and about 50 genera (Schneider & Wynne 2007; Le Gall et al. 2008). Although phylogenetic studies of the Rhodymeniales based on gene sequences are published (Saunders et al. 1999; Le Gall et al. 2008), about 30% of the genera have yet to be analyzed. In addition, a plastid genome for the order has not been deciphered, and only one mitochondrial genome has been announced (*Rhodymenia pseudopalmata* (J.V. Lamouroux) P. C. Silva; GenBank KC875852; Kim et al. 2014). For this study, the marine red algal species *Coeloseira compressa* was analyzed. Hollenberg (1941) originally assigned it to *Coeloseira*, but with a question mark because he lacked cystocarpic material. He noted, however, that the general habit and production of polyspores by *C. compressa* suggested a close relationship to the generitype, *C. parva* G. J. Hollenberg (Hollenberg 1941). According to Hollenberg, *Coeloseira* differed from *Gastroclonium* by the production of polyspores, rather than tetraspores. Smith (1944) and later Abbott and Hollenberg (1976) accepted Hollenberg’s placement of *C. compressa* in *Coeloseira*, but this opinion was rejected by Chang and Xia (1978). The latter transferred *C. compressa* and *C. parva* to *Gastroclonium*, citing that some species of *Gastroclonium*, such as *G. subarticulatum* (Turner) Kützing, possess both polyspores and tetraspores, and that polyspores are derivatives of tetraspores. Hughey (1995), Silva et al. (1996), and Gabrielson et al. (2004) accepted the name *G. compressum* (G.J. Hollenberg) Chang & Xia. Recent anatomical and molecular phylogenetic analyses on the Champiaceae (Le Gall et al. 2008) supported the transfer of *G. subarticulatum* to a new genus, *Neogastroclonium*. *Neogastroclonium* is distinguished from *Gastroclonium* by its solid erect axes and ostiolate cystocarps (Le Gall et al. 2008). Their study, however, did not analyze any species of *Coeloseira*.

To determine the mitochondrial and plastid genome structure of a member of the Champiaceae, and to address the taxonomic status of *Coeloseira*, a specimen of *C. compressa* was analyzed using next generation sequencing techniques. The DNA was extracted from a specimen from Tomales Bay, California (UC 2050599) following the protocol of Lindstrom et al. (2011). The genomic library was constructed and sequenced by the High-Throughput Genomics Center (Seattle, Washington). The data were assembled using Velvet 1.2.08 (Zerbino & Birney 2008) on the Bio-Linux platform (Field et al. 2006) using the assembly and annotation methods described by Hughey et al. (2014). Alignment of the Florideophyte mitogenomes was completed using default settings in MAFFT (Katoh & Standley 2013). The maximum likelihood analysis was performed using RaxML (Stamatakis 2014) with 1000 bootstrap replicates and default parameters in Galaxy (Giardine et al. 2005; Blankenberg et al. 2010; Goecks et al. 2010). The phylogenetic tree was generated with TreeDyn 198.3 at Phylogeny.fr (Dereeper et al. 2008).

The partial mitogenome of *C. compressa* (GenBank KU053956) is 25,391 bp in length, AT rich (74.3%), and contains 46 genes including two ribosomal RNA genes (one rnl and one rns), 20 transfer RNAs, five ribosomal proteins (two rpl and three rps), ymf39 and 18 genes involved in electron transport and oxidative phosphorylation. The mitogenome of *C. compressa* is highly conserved compared to other Florideophyceae (Yang et al. 2015). It differs from *R. pseudopalmata* (GenBank accession no. KC875852) in the tRNAs present in the tandem tRNA region situated between atp6 and secY of the Florideophyte mitogenome. *Coeloseira compressa* contains trnSec, trnY, and a second copy of trnR, whereas *R. pseudopalmata* contains trnM and trnU, which are not present in *C. compressa*. *Coeloseira compressa* also lacks ORF-Rpse35. Phylogenetic analysis of the mitogenome of *C. compressa* resolves it in a fully supported clade with *R. pseudopalmata* in the Rhodymeniales, sister to *Sebdenia flabellata* (J. G. Agardh) P. G. Parkinson and *Grateloupia angusta* (Okamura) S. Kawaguchi & H. W. Wang (Figure 1).

**Figure 1.**
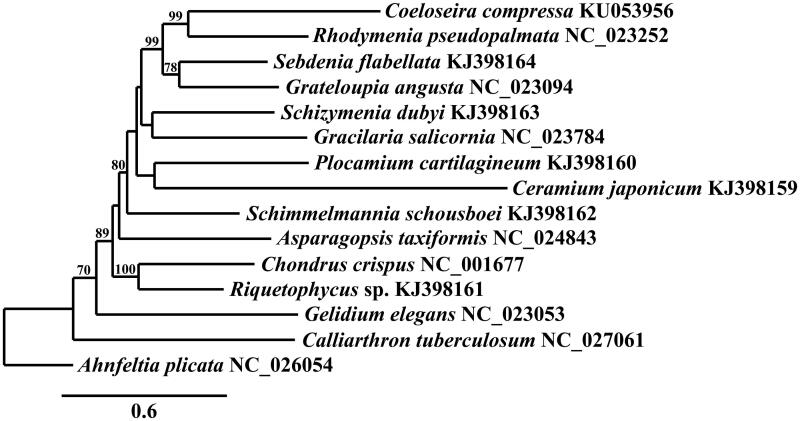
Maximum-likelihood phylogram of representative Florideophyceaen mitogenomes. Numbers along branches are RaxML bootstrap supports based on 1000 nreps (<70% support not shown). The legend represents the scale for nucleotide substitutions.

The complete plastid genome of *C. compressa* (GenBank KU053957) is 176,291 bp in length and contains 233 genes. The genome is AT rich (71.0%), and contains 19 small and 28 large ribosomal proteins, 29 photosystem I and II, 29 tRNA, 33 hypothetical chloroplast reading frames (ycf), 14 Open Reading Frames (ORFs), 10 phycobiliproteins, eight cytochrome b/f complex proteins, eight ATP synthase, three ribosomal RNAs and 62 other genes. Similar to all members of the Rhodymeniophycidae, *C. compressa* contains a single protochlorophyllide reductase gene (chlI). As found in *Gracilaria tenustipitata* var. *liui* Zhang & Xia (GenBank NC_006137; Hagopian et al. 2004), *C. compressa* codes for ycf23 and ycf86, but lacks ycf57.

Phylogenetic analysis of *C. compressa* with other Champiaceae using standard genetic markers (*rbc*L, CO1, and LSU GenBank accession no. KU052799) indicates that *C. compressa* is polyphyletic with respect to the generitype of *Gastroclonium*, *G. ovatum* (Hudson) Papenfuss. Calculation of intergeneric pairwise sequence divergences between representative Champiaceae found that *C. compressa* is most closely related to *N. subarticulatum*. *Coeloseira compressa* differs from *N. subarticulatum* by 5.7% (*rbc*L), 11.0% (CO1) and 1.5% (LSU). These percentages are within interspecific ranges typically reported for red algae, suggesting that *Coeloseira* and *Neogastroclonium* are congeneric. Analysis of the generitype of *Coeloseira* is required to determine the fate of the more recent name *Neogastroclonium*.
